# Remnant cholesterol and severity of nonalcoholic fatty liver disease

**DOI:** 10.1186/s13098-023-01220-9

**Published:** 2023-11-21

**Authors:** Hangkai Huang, Jinghua Wang, Li Wu, Jiaqi Ruan, Linxiao Hou, Chao Shen, Chengfu Xu

**Affiliations:** 1 Department of Gastroenterology, Zhejiang Provincial Clinical Research Center for Digestive Diseases, The First Affiliated Hospital, Zhejiang University School of Medicine, No. 79 Qingchun Road, Hangzhou, 310003 China; 2 Department of Geriatrics, The First Affiliated Hospital, Zhejiang University School of Medicine, Hangzhou, 310003 China; 3 Health Management Center, The First Affiliated Hospital, Zhejiang University School of Medicine, Hangzhou, 310003 China

**Keywords:** Nonalcoholic fatty liver disease, Severity, Remnant cholesterol

## Abstract

**Background:**

Serum remnant cholesterol levels are being increasingly acknowledged as a causal risk factor for atherosclerotic disease, regardless of conventional lipid parameters. The positive association between remnant cholesterol and nonalcoholic fatty liver disease (NAFLD) has been revealed in previous studies. However, whether remnant cholesterol is associated with the severity of NAFLD remains unknown. This study aimed to explore the association between serum remnant cholesterol and the risk of NAFLD severity.

**Methods:**

This cross-sectional study included a total of 6,053 participants who attended health checkups. The severity of hepatic steatosis was evaluated by liver ultrasound transient elastography. Univariable and multivariable logistic regression analyses were performed to calculate the odds ratio (OR) and 95% confidence interval (95% CI) for the association between remnant cholesterol and the severity of hepatic steatosis. To explore whether the association between remnant cholesterol and NAFLD severity was independent of conventional lipid parameters, we further investigated this association in individuals with normal values of low-density lipoprotein-cholesterol (LDL-C), high-density lipoprotein-cholesterol (HDL-C) and triglycerides.

**Results:**

In total, 36.9% of individuals had mild steatosis, and 5.9% had moderate-to-severe steatosis. The serum level of remnant cholesterol in nonsteatosis, mild steatosis and moderate-to-severe steatosis gradually increased (0.71 ± 0.33, 0.97 ± 0.52 and 1.07 ± 0.63 mmol/L, respectively). In the multivariable mode, remnant cholesterol was positively associated with mild hepatic steatosis (OR: 1.730, 95% CI: 1.541 − 1.941, *P* < 0.001) and moderate-to-severe steatosis (OR: 2.342, 95% CI: 1.765 − 3.109, *P* < 0.001). These associations were not significantly altered in individuals with normal triglycerides, HDL-C and LDL-C (OR: 1.664, 95% CI: 1.448 − 1.911, *P* < 0.001; OR: 2.269, 95% CI: 1.619 − 3.180, *P* < 0.001, respectively).

**Conclusions:**

Higher levels of serum remnant cholesterol were associated with more severe hepatic steatosis, regardless of conventional lipid parameters. Individuals with higher remnant cholesterol may need more attention in regular surveillance of NAFLD.

## Introduction

Nonalcoholic fatty liver disease (NAFLD) is the leading cause of chronic liver disease worldwide [1]. The pooled prevalence of NAFLD was estimated to be nearly 30% [2]. NAFLD encompasses a spectrum of diseases, progressing from simple steatosis to steatohepatitis, fibrosis and cirrhosis [3]. Patients with NAFLD are at higher risk for a range of hepatic and nonhepatic long-term outcomes [4]. NAFLD was positively associated with the risk of ischemic heart disease, stroke and congestive heart failure [5]. Furthermore, the risk of major adverse cardiovascular events gradually increased with the severity of NAFLD, with hazard ratios for steatosis, fibrosis and cirrhosis of 1.58, 1.67 and 2.15, respectively [5]. Patients with NAFLD carry higher risks of all-cause mortality and cardiovascular and liver-related mortality, which increase with the progression of NAFLD [6]. The clinical outcomes of NAFLD vary according to its severity [4]. Early identification of more severe NAFLD patients may contribute to preventing adverse outcomes of NAFLD [7]. Epidemiological studies are needed to provide more information on the severity of NAFLD.

Lowering the serum levels of low-density lipoprotein-cholesterol (LDL-C) mainly through statins is the mainstream therapy target for the secondary prevention of cardiovascular disease (CVD) [8]. However, even when LDL-C is substantially reduced and already in the optimal range, patients still have a considerable risk of atherosclerosis [9]. Recent research attention has shifted to identifying indicators predictive of this residual risk [10]. Remnant cholesterol refers to the cholesterol content carried by triglyceride-rich lipoproteins, which include non-LDL-C and nonhigh-density lipoprotein-cholesterol (HDL-C) [11]. A large number of studies have reported that serum remnant cholesterol levels were positively associated with the risk of coronary artery disease [12], NAFLD [13], type 2 diabetes [14] and metabolic syndrome [15]. Previous studies found that remnant cholesterol was independently associated with the risk of NAFLD in the general population [16], nonobese adults [17] and adolescents [18]. The different severities of NAFLD have vastly different long-term outcomes [19]. Studies of NAFLD should stratified according to severity. To date, whether the serum levels of remnant cholesterol are associated with the severity of NAFLD remains unclear.

This study aimed to explore the association between remnant cholesterol and the severity of NAFLD. In addition, we explored whether this association was independent of traditional lipid profiles.

## Methods

### Study population

This cross-sectional study enrolled adults who attended health checkups at the First Affiliated Hospital, Zhejiang University School of Medicine between 2021 and 2022. Exclusion criteria included (i) those without complete demographic, laboratory and liver ultrasound transient elastography data; (ii) drinking heavily defined as alcohol intake more than 210 g/week for males or 140 g/week for females; (iii) having a history of viral hepatitis, autoimmune hepatitis or other chronic liver disease; and (iv) taking antihyperlipidemic agents. A total of 6,053 participants were included in this study. This study was approved by the Hospital Ethics Committee (IIT20230490).

### Clinical measurements

Details of the clinical measurements have been described elsewhere [20, 21]. Body weight was measured in light clothes, and standing height was measured with shoes removed. We defined smokers as having smoked at least 100 cigarettes in their lifetime [22]. Blood pressure was checked by electronic sphygmomanometers after resting for five minutes. Overnight fasting venous blood samples were collected from all participants. Serum levels of uric acid, γ-glutamyl transpeptidase, total cholesterol, LDL-C, HDL-C, triglyceride, glucose and glycated hemoglobin A1c were detected by a Hitachi 7600 autoanalyzer (Hitachi, Tokyo, Japan) or a Sysmex XE-2100 autoanalyzer (Sysmex, Kobe, Japan). Remnant cholesterol was calculated as total cholesterol minus HDL-C minus LDL-C [23].

The normal range of triglycerides was defined as < 1.69 mmol/L [24]. LDL-C ≥ 2.59 mmol/L was recognized as over the normal limit [24]. Decreased HDL-C was identified as < 1.03 mmol/L for males or < 1.29 mmol/L for females [25]. The clinical cutoff point for non-HDL-C was 3.37 mmol/L [24]. We diagnosed hypertension as blood pressure ≥ 140/90 mmHg or taking antihypertensive agents [26]. The definition of diabetes was fasting plasma glucose ≥ 7 mmol/L, glycated hemoglobin A1c ≥ 6.5% or taking hypoglycemic medications [27].

### Evaluation of NAFLD severity

NAFLD was diagnosed as the presence of fatty liver without other causes of chronic liver disease [28]. Fatty liver was measured by liver ultrasound transient elastography using the FibroScan Model 502 V2 Touch equipped with a medium (M) or extralarge (XL) probe. We identified hepatic steatosis with controlled attenuation parameters ≥ 261 dB/m [29]. Moderate-to-severe hepatic steatosis was defined as having controlled attenuation parameters ≥ 305 dB/m. Mild steatosis was defined as having a controlled attenuation parameter of 261 − 305 dB/m.

### Statistical analyses

Variables are shown as the mean (standard deviation), median (interquartile range) or as proportions. Comparisons among groups were determined by ANOVA, Kruskal‒Wallis rank-sum test or chi-square test. We conducted univariable and multivariable logistic regression analyses to explore the association between remnant cholesterol and NAFLD severity. Covariates adjusted for in the multivariable model included age, sex, body mass index (BMI), systolic and diastolic blood pressure, smoking status, fasting plasma glucose, glycated hemoglobin A1c, γ-glutamyl transpeptidase, total cholesterol and LDL-C. To explore whether the association between remnant cholesterol and NAFLD severity was independent of conventional lipid parameters, we further investigated this association in individuals with normal values of LDL-C, HDL-C and triglycerides. In addition, we compared the risk of NAFLD severity across LDL-C vs. remnant cholesterol discordant/concordant groups. The percentile equivalents of remnant cholesterol were calculated according to clinical cut-points of LDL-C. Participants were stratified into four groups: low LDL-C and low remnant cholesterol, low LDL-C and high remnant cholesterol, high LDL-C and low remnant cholesterol, and high LDL-C and high remnant cholesterol. Using the first group as a reference, the risk of mild and moderate-to-severe steatosis was estimated for the latter three groups. The risk of NAFLD severity was also compared across non-HDL-C vs. remnant cholesterol discordant/concordant groups. In subgroup analyses, participants were grouped according to age, sex, BMI, diabetes and hypertension. All analyses were conducted with SAS version 9.4 (SAS Institute, Cary, NC). A *P* value < 0.05 (two-tailed) was considered significant.

## Results

### Clinical features of participants

Table 1 depicts the clinical features of participants stratified by the quartiles of remnant cholesterol. Individuals with higher remnant cholesterol had higher BMI, systolic blood pressure, diastolic blood pressure, serum uric acid, total cholesterol, LDL-C, triglyceride, glucose, glycated hemoglobin A1c and γ-glutamyl transpeptidase. They were more likely to be male, smoking and old and had higher proportions of mild hepatic steatosis (from Q1 to Q4: 21.29, 32.14, 40.50 and 54.14%, respectively) and moderate-to-severe steatosis (from Q1 to Q4: 1.89, 3.64, 6.42 and 11.68%, respectively). The value of remnant cholesterol gradually increased in participants with no steatosis, mild steatosis and moderate-to-severe steatosis (0.71 ± 0.33 mmol/L, 0.96 ± 0.52 mmol/L and 1.10 ± 0.60 mmol/L, respectively).

**Table 1:** Clinical characteristics of participants stratified by quartiles of remnant cholesterol

Variables	Quartile 1 (*n* = 1,536)	Quartile 2 (*n* = 1,540)	Quartile 3 (*n* = 1,479)	Quartile 4 (*n* = 1,498)	*P* value
Female, %	56.38	46.04 ^*^	36.58 ^† §^	28.50 ^‡ || ¶^	< 0.001
Age, year	49.69 (12.51)	50.96 (11.31) ^*^	51.53 (10.42) ^† §^	52.07 (10.02) ^‡ || ¶^	< 0.001
Body mass index, kg/m^2^	22.64 (2.94)	23.73 (3.01) ^*^	24.56 (2.97) ^† §^	25.55 (3.01) ^‡ || ¶^	< 0.001
Current smoker, %	13.48	16.88 ^*^	24.75 ^† §^	30.77 ^‡ || ¶^	< 0.001
Systolic blood pressure, mmHg	117.93 (16.83)	122.62 (17.29) ^*^	123.92 (16.79) ^† §^	126.11 (16.08) ^‡ || ¶^	< 0.001
Diastolic blood pressure, mmHg	69.55 (11.13)	72.95 (11.11) ^*^	73.95 (11.03) ^† §^	75.98 (10.72) ^‡ || ¶^	< 0.001
Serum uric acid, µmol/L	289.03 (71.58)	318.65 (80.43) ^*^	339.63 (82.40) ^† §^	367.94 (83.28) ^‡ || ¶^	< 0.001
Total cholesterol, mmol/L	4.29 (0.77)	4.66 (0.76) ^*^	5.00 (0.84) ^† §^	5.41 (0.97) ^‡ || ¶^	< 0.001
Triglyceride, mmol/L	0.83 (0.25)	1.16 (0.33) ^*^	1.56 (0.43) ^† §^	2.88 (1.86) ^‡ || ¶^	< 0.001
HDL-C, mmol/L	1.51 (0.36)	1.33 (0.32) ^*^	1.20 (0.28) ^† §^	1.03 (0.24) ^‡ || ¶^	< 0.001
LDL-C, mmol/L	2.36 (0.62)	2.68 (0.62) ^*^	2.95 (0.71) ^† §^	3.00 (0.87) ^‡ || ¶^	< 0.001
Fasting plasma glucose, mmol/L	4.75 (0.83)	4.92 (1.03) ^*^	5.00 (1.18) ^† §^	5.23 (1.56) ^‡ || ¶^	< 0.001
Glycated hemoglobin A1c, %	5.64 (0.59)	5.74 (0.74) ^*^	5.82 (0.76) ^† §^	5.97 (1.03) ^‡ || ¶^	< 0.001
Remnant cholesterol, mmol/L	0.43 (0.10)	0.65 (0.05) ^*^	0.85 (0.07) ^† §^	1.37 (0.56) ^‡ || ¶^	< 0.001
γ-glutamyl transpeptidase, U/L	15.00 [12.00, 22.00]	19.00 [14.00, 28.00] ^*^	24.00 [16.00, 36.00] ^† §^	32.00 [21.00, 51.00] ^‡ || ¶^	< 0.001
Severity of hepatic steatosis, %					< 0.001
Mild steatosis	21.29	32.14 ^*^	40.50 ^† §^	54.14 ^‡ || ¶^	
Moderate-to-severe steatosis	1.89	3.64 ^*^	6.42 ^† §^	11.68 ^‡ || ¶^	
Overall steatosis	23.18	35.78 ^*^	46.92 ^† §^	65.82 ^‡ || ¶^	

Data are presented as the mean (standard deviation), median (interquartile range) or as proportions

HDL-C, high density lipoprotein cholesterol; LDL-C, low density lipoprotein cholesterol

*P* value: statistical significance among the four groups; ^*^ Statistical significance with Bonferroni correction for measurement data between Quartile 1 and Quartile 2, ^†^ Quartile 1 versus Quartile 3, ^‡^ Quartile 1 versus Quartile 4, ^§^ Quartile 2 versus Quartile 3, ^||^ Quartile 2 versus Quartile 4, ^|¶^ Quartile 3 versus Quartile 4

### Association between remnant cholesterol and NAFLD severity

**Fig. 1:**
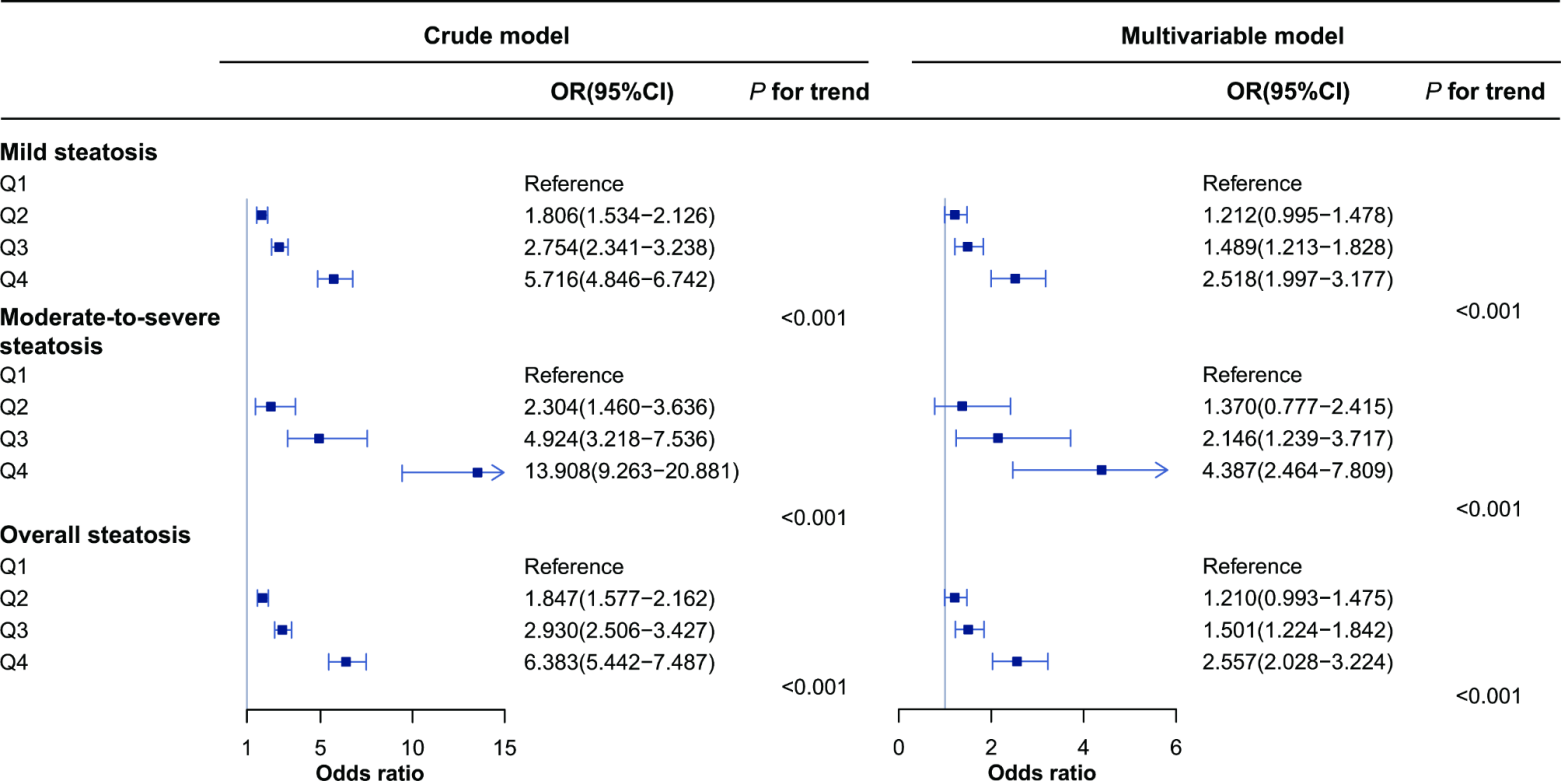
Association of remnant cholesterol with severity of hepatic steatosis. Remnant cholesterol in quartiles 1 to 4 was ≤ 0.55, 0.56 − 0.74, 0.75 − 0.97, and > 0.97 mmol/L, respectively. The multivariable model was adjusted for age, sex, BMI, systolic blood pressure, diastolic blood pressure, smoking, fasting plasma glucose, glycated hemoglobin A1c, γ-glutamyl transpeptidase, low-density lipoprotein-cholesterol and total cholesterol

In the multivariable logistic regression model, the serum level of remnant cholesterol was positively associated with mild hepatic steatosis and more strongly associated with moderate-to-severe steatosis (_ORQ4 vs. Q1_: 2.518, 95% CI: 1.997 − 3.117; _ORQ4 vs. Q1_: 4.387, 95% CI: 2.464 − 7.809, respectively) (Fig. 1). With the increase in remnant cholesterol, the risk of mild as well as moderate-to-severe hepatic steatosis gradually increased (all *P* for trend < 0.001). The odds ratios for mild and moderate-to-severe steatosis were estimated to be 1.730 (95% CI: 1.541 − 1.941) and 2.342 (95% CI: 1.765 − 3.109), respectively, with a 1 standard deviation increase in remnant cholesterol (Table 2).

**Table 2:** Association of per 1 standard deviation increase in remnant cholesterol with severity of hepatic steatosis

	Crude model	Multivariable model
	OR (95% CI)	OR (95% CI)
Mild steatosis	2.237 (2.074 to 2.414)***	1.730 (1.541 to 1.941)***
Moderate-to-severe steatosis	2.620 (2.373 to 2.892)***	2.342 (1.765 to 3.109)***
Overall steatosis	2.287 (2.121 to 2.465)***	1.743 (1.553 to 1.956)***

The multivariable model was adjusted for age, sex, BMI, systolic blood pressure, diastolic blood pressure, smoking, fasting plasma glucose, glycated hemoglobin A1c, γ-glutamyl transpeptidase, low-density lipoprotein-cholesterol and total cholesterol

* *P* < 0.05, ** *P* < 0.005, *** *P* < 0.001

**Fig. 2:**
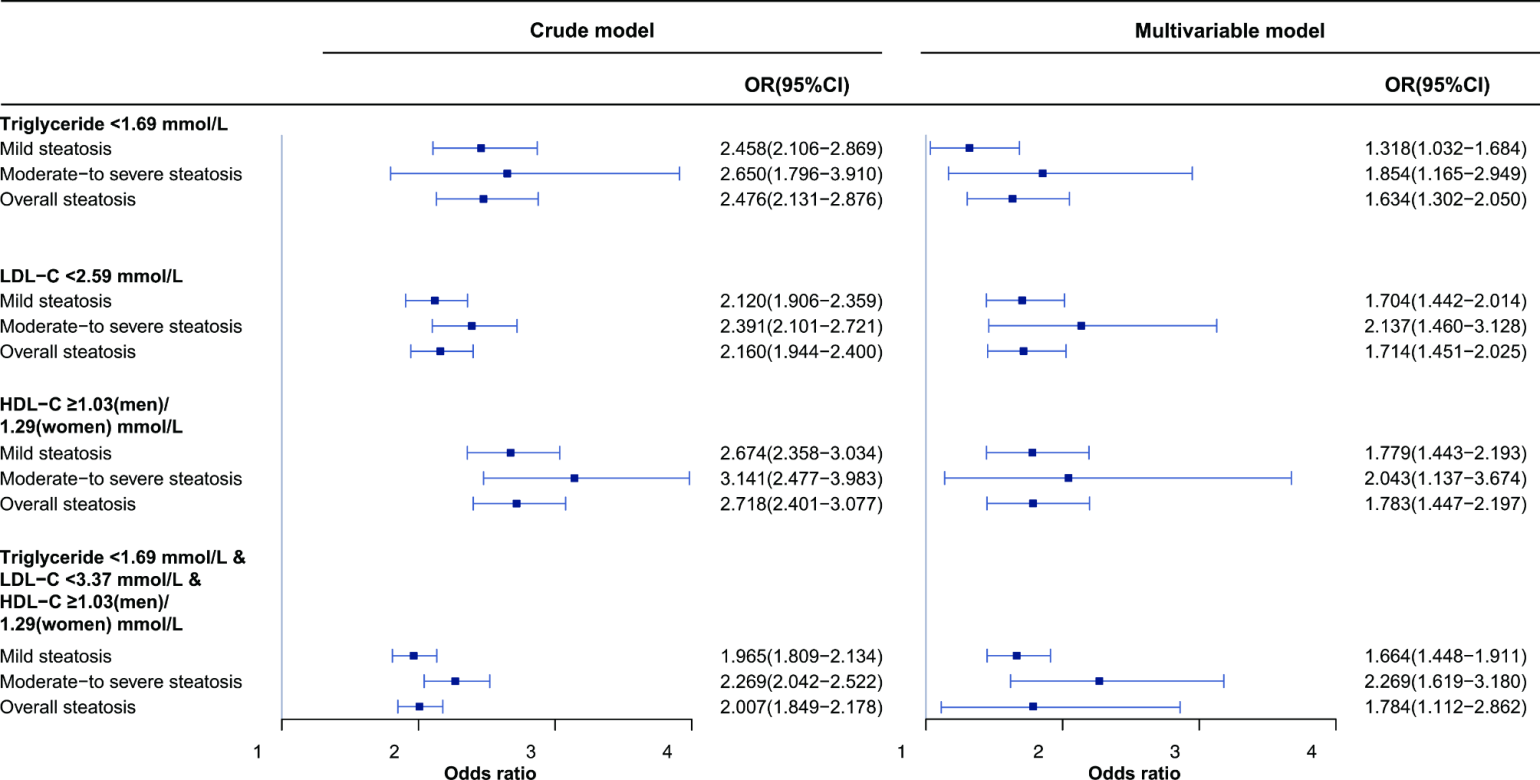
Association between per 1 standard deviation increase in remnant cholesterol and severity of hepatic steatosis in participants with normal levels of serum triglyceride, LDL-C, and HDL-C. HDL-C, high-density lipoprotein cholesterol, LDL-C, low-density lipoprotein cholesterol The multivariable model was adjusted for age, sex, BMI, systolic blood pressure, diastolic blood pressure, smoking, fasting plasma glucose, glycated hemoglobin A1c, γ-glutamyl transpeptidase, low-density lipoprotein-cholesterol and total cholesterol

We further explored the association between remnant cholesterol and NAFLD severity among subjects with normal ranges of conventional lipid indicators (Fig. 2). We observed that remnant cholesterol showed a stronger association with moderate-to-severe steatosis than with mild steatosis in those with normal triglycerides, LDL-C and HDL-C. Even among individuals with all these parameters in the optimal range, the odds ratio for moderate-to-severe steatosis (OR: 2.269, 95% CI: 1.619 − 3.180) was greater than that for mild steatosis (OR: 1.664, 95% CI: 1.448 − 1.911).

In addition, we compared the risk of NAFLD across LDL-C and remnant cholesterol concordant/discordant groups (Table 3). Compared with the concordant low LDL-C/remnant cholesterol group, the discordant low LDL-C/high remnant cholesterol group and concordant high LDL-C/remnant cholesterol group showed an increased risk of mild hepatic steatosis (OR: 1.770, 95% CI: 1.425 − 2.199; OR: 1.609, 95% CI: 1.252 − 2.067, respectively), while the discordant high LDL-C/low remnant cholesterol group did not (OR: 1.059, 95% CI: 0.882 − 1.364). This phenomenon was also observed for moderate-to-severe steatosis.

**Table 3:** Odds ratios (95% confidence interval) for NAFLD severity across LDL-C vs. remnant cholesterol concordant/discordant groups by LDL-C clinical cut-points and percentile equivalents for remnant cholesterol

	Crude model	Multivariable model
	OR (95% CI)	OR (95% CI)
**Mild steatosis**		
LDL-C < cut-point & RC < cut-point	Reference	Reference
LDL-C < cut-point & RC ≥ cut-point	3.373 (2.841 to 4.005)***	1.770 (1.425 to 2.199)***
LDL-C ≥ cut-point & RC < cut-point	1.060 (0.888 to 1.266)	1.059 (0.882 to 1.364)
LDL-C ≥ cut-point & RC ≥ cut-point	2.858 (2.477 to 3.298)***	1.609 (1.252 to 2.067)***
**Moderate-to-severe steatosis**		
LDL-C < cut-point & RC < cut-point	Reference	Reference
LDL-C < cut-point & RC ≥ cut-point	5.874 (4.126 to 8.362)***	2.576 (1.596 to 4.160)***
LDL-C ≥ cut-point & RC < cut-point	0.554 (0.324 to 1.289)	0.913 (0.451 to 1.849)
LDL-C ≥ cut-point & RC ≥ cut-point	3.763 (2.712 to 5.221)***	1.842 (1.033 to 3.287)*

The clinical cutoffoff points for LDL-C and remnant cholesterol were 2.59 mmol/L and 0.70 mmol/L, respectively

The multivariable model was adjusted for age, sex, BMI, systolic blood pressure, diastolic blood pressure, smoking, fasting plasma glucose, glycated hemoglobin A1c, γ-glutamyl transpeptidase, low-density lipoprotein-cholesterol and total cholesterol

* *P* < 0.05, ** *P* < 0.005, *** *P* < 0.001

We next estimated the odds of mild steatosis across non-HDL-C and remnant cholesterol concordant/discordant groups (Table 4). A positive association was found in the discordant low non-HDL-C/high remnant cholesterol (OR: 1.499, 95% CI: 1.193 − 1.883) and concordant high non-HDL-C/remnant cholesterol groups (OR: 2.003, 95% CI: 1.615 − 2.484) but not in the discordant high non-HDL-C/low remnant cholesterol group (OR: 1.314, 95% CI: 0.992 − 3.095). The risk of moderate-to-severe steatosis showed the same trend.

**Table 4:** Odds ratios (95% confidence interval) for severity of hepatic steatosis across non-HDL-C vs. remnant cholesterol concordant/discordant groups by non-HDL-C clinical cut-points and percentile equivalents for remnant cholesterol

	Crude model	Multivariable model
	OR (95% CI)	OR (95% CI)
**Mild steatosis**		
Non-HDL-C < cut-point & RC < cut-point	Reference	Reference
Non-HDL-C < cut-point & RC ≥ cut-point	2.752 (2.280 to 3.321)***	1.499 (1.193 to 1.883)***
Non-HDL-C ≥ cut-point & RC < cut-point	1.295 (0.995 to 1.567)	1.314 (0.992 to 3.095)
Non-HDL-C ≥ cut-point & RC ≥ cut-point	3.282 (2.877 to 3.744)***	2.003 (1.615 to 2.484)***
**Moderate-to-severe steatosis**		
Non-HDL-C < cut-point & RC < cut-point	Reference	Reference
Non-HDL-C < cut-point & RC ≥ cut-point	3.998 (2.669 to 5.990)***	1.844 (1.099 to 3.095)*
Non-HDL-C ≥ cut-point & RC < cut-point	0.783 (0.437 to 1.400)	1.645 (0.792 to 3.414)
Non-HDL-C ≥ cut-point & RC ≥ cut-point	5.347 (3.931 to 7.272)***	3.365 (2.025 to 5.594)***

The clinical cutoffoff points for non-HDL-C and remnant cholesterol were 3.37 mmol/L and 0.70 mmol/L, respectively

The multivariable model was adjusted for age, sex, BMI, systolic blood pressure, diastolic blood pressure, smoking, fasting plasma glucose, glycated hemoglobin A1c, γ-glutamyl transpeptidase, low-density lipoprotein-cholesterol and total cholesterol

* *P* < 0.05, ** *P* < 0.005, *** *P* < 0.001

### Subgroup analyses

**Fig. 3:**
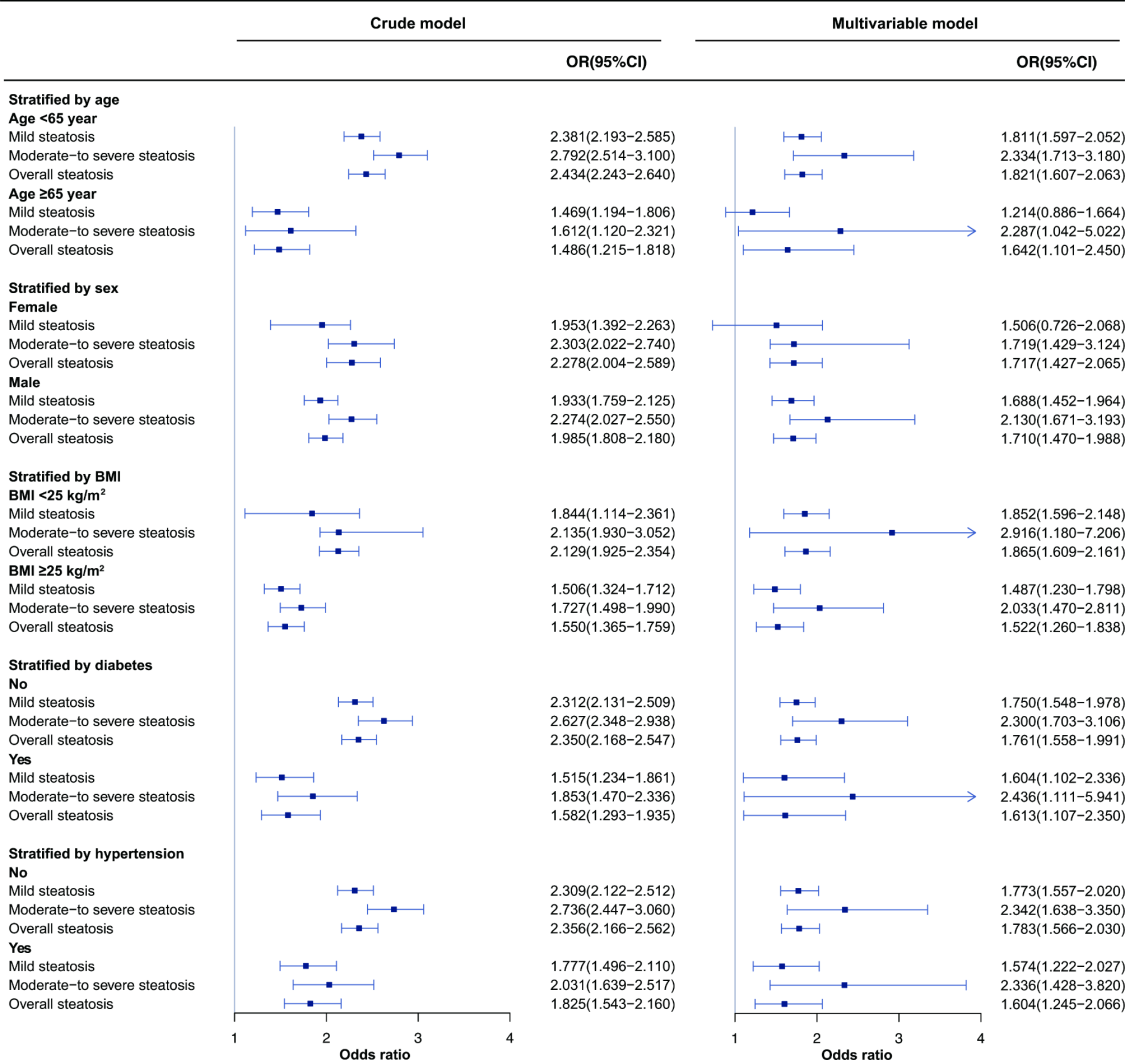
Subgroup analysis of the association between per 1 standard deviation increase in remnant lipoprotein cholesterol and severity of hepatic steatosis. The multivariable model was adjusted for age, sex, BMI, systolic blood pressure, diastolic blood pressure, smoking, fasting plasma glucose, glycated hemoglobin A1c, γ-glutamyl transpeptidase, low-density lipoprotein-cholesterol and total cholesterol

We conducted subgroup analyses stratified by age, sex, BMI, diabetes and hypertension (Fig. 3). The stronger association of remnant cholesterol with moderate-to-severe steatosis than with mild steatosis did not differ by subgroup analyses. The serum level of remnant cholesterol was positively associated with mild hepatic steatosis and more strongly associated with moderate-to-severe steatosis both in subjects with BMI < 25 km/m^2^ (OR: 1.852, 95% CI: 1.596 − 2.148; OR: 2.916, 95% CI: 1.180 − 7.206, respectively) and with BMI ≥ 25 km/m^2^ (OR: 1.487, 95% CI: 1.230 − 1.798; OR: 2.033, 95% CI: 1.470 − 2.811, respectively), both in subjects without diabetes (OR: 1.750, 95% CI: 1.548 − 1.978; OR: 2.300, 95% CI: 1.703 − 3.106, respectively) and with diabetes (OR: 1.604, 95% CI: 1.102 − 2.336; OR: 2.436, 95% CI: 1.111 − 5.941, respectively), and both in subjects without hypertension (OR: 1.773, 95% CI: 1.557 − 2.020; OR: 2.342, 95% CI: 1.638 − 3.350, respectively) and with hypertension (OR: 1.574, 95% CI: 1.222 − 2.027; OR: 2.336, 95% CI: 1.428 − 3.820, respectively).

## Discussion

This study observed that serum remnant cholesterol was associated with increased severity of NAFLD. Notably, this association was independent of traditional lipid profiles. These findings suggested that subjects with higher remnant cholesterol are at higher risk for more severe NAFLD and may need more attention in regular surveillance of NAFLD.

Substantial observational studies have demonstrated that remnant cholesterol is associated with CVD risks [30]. A retrospective cohort study of 17,532 CVD-free individuals showed that remnant cholesterol was associated with a 1.65-fold increased risk of developing CVD during 18.7 years of follow-up [23]. Another cohort study of 6,723 patients with coronary artery disease found that the highest quartile of remnant cholesterol was associated with a 1.9-fold risk of recurrent cardiovascular events compared with the lowest quartile [31]. A Danish population-based cohort showed that adults with remnant cholesterol ≥ 1 mmol/L were 2.2 times more likely to die from CVD [32]. The predictive value of remnant cholesterol was also observed in other cardiometabolic diseases. In a cohort study of nearly half a million adults, the hazard ratio for incident type 2 diabetes was 1.95 in the fourth quartile of remnant cholesterol compared with the first quartile [14]. In a Chinese cohort study of 4,237 individuals, remnant cholesterol was significantly associated with the risk of diabetic nephropathy with a hazard ratio of 1.211 [33].

In addition, the serum level of remnant cholesterol has been independently associated with NAFLD [13]. During 5 years of follow-up, adults with the highest quartile of remnant cholesterol carried a 2.8-fold risk of developing NAFLD [16]. A retrospective study of 6,634 Chinese adults with 43 months of follow-up reported that remnant cholesterol was associated with a 1.143-fold risk of incident NAFLD [34]. A cross-sectional study of 3,370 adults found that there was a nonlinear association between remnant cholesterol and NAFLD [35]. In a study of 1,170 adolescents, the hazard ratios for ultrasonography-diagnosed NAFLD in males and females with the lowest quartile of remnant cholesterol were 0.15 and 0.44, respectively [18]. This study also found that the serum level of remnant cholesterol gradually increased in adolescents with nonsteatosis, mild steatosis and moderate-to-severe steatosis. However, they did not investigate the association between remnant cholesterol and NAFLD severity. In the present study, we observed this phenomenon and further explored this association. We found that increased remnant cholesterol was associated with increased severity of NAFLD. Of note, this association was independent of LDL-C, HDL-C and triglycerides. These findings suggested that individuals with higher remnant cholesterol are at higher risk for more severe NAFLD. Given that more severe NAFLD was related to more adverse clinical outcomes, the routine monitoring of remnant cholesterol may provide more opportunities for reducing poor outcomes of NAFLD. This speculation still needs to be verified in future studies.

This study had several limitations. First, this was a cross-sectional study, and we were unable to draw conclusions about the causal relationship between remnant cholesterol and NAFLD severity. Second, the severity of hepatic steatosis in this study was evaluated by transient elastography rather than liver biopsy. Liver biopsy may provide more histological information, including inflammation and fibrosis. Third, remnant cholesterol was calculated based on standard biochemistry profiles but not direct measurement. However, the calculation of remnant cholesterol is practical and economical, and there is a close correlation between the calculated value and the measured value [36].

## Conclusion

In conclusion, higher serum levels of remnant cholesterol were associated with more severe hepatic steatosis. This association was not significantly changed in individuals with the optimal ranges of triglycerides, HDL-C and LDL-C. These results indicated that individuals with higher remnant cholesterol are at higher risk for more severe NAFLD, regardless of their traditional lipid profiles. This needs to be validated in future studies.

## Data Availability

The datasets used and analyzed during the current study are available from the corresponding author upon reasonable request.
